# Integrated Transcriptomics and Network Pharmacology Reveal the Mechanism of Poplar-Type Propolis on the Mouse Mastitis Model

**DOI:** 10.3390/nu17233683

**Published:** 2025-11-25

**Authors:** Meifei Zhu, Ruike Wei, Bin Yuan, Shanshan Li, Fuliang Hu

**Affiliations:** College of Animal Sciences, Zhejiang University, Hangzhou 310058, China; meifeizhu@zju.edu.cn (M.Z.); wrk0326@zju.edu.cn (R.W.); yuan_bin322@zju.edu.cn (B.Y.); flhu@zju.edu.cn (F.H.)

**Keywords:** propolis, mastitis, blood–milk barrier, chrysin, CAPE, galangin, NF-κB, TNF, JAK-STAT

## Abstract

Background/Objectives: Mastitis adversely affects human lactation, and there is a need for effective natural therapeutic agents. Poplar-type propolis is known for its anti-inflammatory properties, but its protective effects and mechanisms against mastitis remain unclear. This study aimed to investigate the therapeutic potential and underlying mechanisms of ethanol extract of Chinese propolis (EECP) against lipopolysaccharide (LPS)-induced mastitis. Methods: An integrated approach combining network pharmacology and transcriptomics was employed. In vivo validation was conducted using an LPS-induced mouse mastitis model in female BALB/c mice. Molecular docking was used to confirm interactions between key EECP components and core targets. Results: Network pharmacology identified 36 potential targets, primarily involved in inflammatory and immune pathways such as tumor necrosis factor (TNF), nuclear factor kappa B (NF-κB), janus kinase-signal transducers and activators of transcription (JAK-STAT), phosphatidylinositol 3-kinase/protein kinase B (PI3K-AKT), and interleukin (IL)-17 pathways. In vivo experiments demonstrated that EECP significantly alleviated LPS-induced histopathological damage, reduced neutrophil infiltration, and decreased the expression of proinflammatory cytokines (*TNFα*, *IL1β*, and *IL6*). Furthermore, EECP restored the expression and distribution of tight junction proteins (ZO-1 and occludin), thereby preserving blood–milk barrier integrity. Transcriptomic analysis confirmed that EECP reversed LPS-induced gene expression changes and downregulated key inflammation-related pathways, including TNF, NF-κB, JAK-STAT, and IL-17. Integrated analysis identified *TNF*, *IL6*, *IL1B*, interferon gamma *(IFNG)*, *STAT3*, and *CXCL8* as core targets. Molecular docking confirmed strong binding interactions between characteristic propolis polyphenols (e.g., chrysin, CAPE, and galangin) and these core targets. Conclusions: EECP exerts protective effects against LPS-induced mastitis through the synergistic actions of multiple components. This study lays the preclinical foundation for considering poplar-type propolis as a candidate for the prevention or alleviation of mastitis, meriting further evaluation.

## 1. Introduction

Mastitis, an inflammatory condition of the mammary gland, is predominantly caused by bacterial infection (e.g., *Escherichia coli* and *Staphylococcus aureus*) and represents a major challenge in human lactation [1]. Lipopolysaccharide (LPS), a key component of the outer membrane of Gram-negative bacteria, is widely used to induce experimental mastitis models due to its ability to trigger robust inflammatory responses [2]. The pathogenesis of mastitis is driven by specific molecular events, primarily the activation of the TLR4/NF-κB and JAK-STAT signaling pathways upon bacterial challenge. This leads to a storm of proinflammatory cytokines (e.g., TNF-α, IL-6, and IL-1β) and the disruption of tight junctions, critically compromising blood–milk barrier integrity [3]. A systematic review has indicated that mastitis affects 25% of lactating women during the first 26 weeks postpartum [4]. In the absence of appropriate and timely management, mastitis can lead to severe complications such as breast abscesses and septic shock [5]. Current treatments often rely on antibiotics, which raise concerns about residues and antimicrobial resistance [6]. Therefore, identifying natural anti-inflammatory agents with minimal side effects is of great interest.

Propolis, a resinous substance collected by honeybees from plant buds and exudates, has been used traditionally for its broad-spectrum biological activities, including anti-inflammatory, antioxidant, and antimicrobial properties [7]. Poplar-type propolis, sourced from subtropical or temperate regions, including Europe, North America, and Asia, is rich in flavonoids and phenolic acids [8]. It has demonstrated potential in modulating immune responses and tissue repair [9]. While a limited number of studies have suggested the potential of propolis against mastitis, the evidence remains fragmented and predominantly preliminary. In vitro studies have demonstrated its pronounced antimicrobial activity against mastitis-associated pathogens such as *Staphylococcus* spp. [10]. Furthermore, formulations like propolis-based emulgels have been explored for topical application and showed promise in alleviating clinical symptoms [11]. However, in vivo investigations, like those utilizing established animal models to elucidate the systemic protective mechanisms, are strikingly scarce. Consequently, the underlying mechanisms governing its anti-inflammatory and barrier-protective effects in mastitis remain largely unresolved.

To address this issue and elucidate the complex mechanism underlying propolis, a systems-level approach is essential. The integrated strategy combining network pharmacology with transcriptomics is particularly well-suited for this purpose. In recent years, the research methodology that merges network pharmacology with transcriptomics has emerged as an effective method to explore the pharmacodynamic mechanisms of traditional Chinese medicine (TCM), owing to its remarkable ability to capture both macro and micro-level interactions [12]. The network pharmacology uses databases to predict potential targets and associated pathways of TCMs [13], while transcriptomics employs high-throughput sequencing on biological samples to assess transcriptional regulation under real experimental conditions. The common targets identified through both network pharmacology and transcriptomics may be regarded as potential core targets.

Given the complexity and diversity of its components, elucidating the mechanism of propolis presents a significant challenge. To address this, we employed an integrated network pharmacology and transcriptomics strategy. Within this framework, and informed by prior evidence of its anti-inflammatory properties, we hypothesized that the ethanol extract of Chinese propolis (EECP) alleviates mastitis through the multi-target, cooperative suppression of the NF-κB, JAK-STAT, and IL-17 signaling pathways.

## 2. Materials and Methods

### 2.1. Reagents

Poplar-type propolis was provided by the Flavormax Nutrition Co., Ltd. (Shanghai, China). DEX and LPS (*E. coli* O111:B4) was obtained from Sigma-Aldrich (St. Louis, MO, USA). The primary antibodies ZO-1 and Occludin were obtained from Abcam (Cambridge, UK).

### 2.2. Preparation of Poplar-Type Propolis

Following a previously established method [14], raw propolis was ground and sieved through a 20-mesh screen. Ten grams of the resulting powder were added to 100 mL of 95% (*v/v*) ethanol in a beaker. The mixture was then extracted at 40 °C for 24 h with gentle stirring. Subsequently, the extract was centrifuged at 8800 rpm for 10 min. Thereafter, the supernatant was collected and stored at −20 °C overnight. The cooled solution was subsequently filtered through Whatman filter paper to remove particulates. The filtered extract was concentrated by rotary evaporation and then freeze-dried. The final propolis extraction powder was collected for further analysis.

### 2.3. Ethics Statement

All animal procedures were approved by the Institutional Animal Care and Use Committee of Zhejiang Chinese Medical University (IACUC-202304-10) and implemented according to the guidelines of the Laboratory Animal Research Center of Zhejiang Chinese Medical University (Certificate No. SYXK, Zhejiang, 2021-0012, China).

### 2.4. Mice and Treatments

BALB/c mice (8–9 weeks old, 20–25 g) were sourced from the Animal Experimental Center of Zhejiang Chinese Medical University (Hangzhou, China). They were housed under standard conditions (22 °C–23 °C, 12 h light–dark cycle) with two females and one male per cage. Pregnant females were subsequently separated into individual cages. Twenty-four nursing mice were randomly assigned to four groups (*n* = 6): Control (CK), LPS (0.2 mg/mL, 50 µL), LPS+ ethanol extract of Chinese propolis (EECP, 100 mg/kg/d), and LPS+ dexamethasone (DEX) (5 mg/kg/d). The dosages of LPS [15] and EECP [16] were determined based on previous studies. The administration of EECP and DEX was conducted via gavage for five days prior to the initiation of LPS treatment, which occurred from days 5 to 9 of lactation, while the CK and LPS groups received an equivalent volume of sterile saline. On day 9, one hour after the final gavage, mastitis was induced in all except the CK group by an intraductal injection of LPS into the fourth mammary glands using a microsyringe with a 32G needle. All mice were euthanized 24 h post-LPS injection for mammary tissue collection.

### 2.5. Hematoxylin−Eosin (H&E) Staining

The mammary gland tissues were immersed in a 4% paraformaldehyde solution for over 24 h to achieve fixation. Following this, the tissues were embedded in paraffin and sectioned into pieces measuring 5 μm in thickness. To evaluate histological alterations, the sections were stained with haematoxylin–eosin and examined under a microscope (NIKON ECLIPSE CI, Nikon, Tokyo Metropolis, Japan). Histological sections were assessed using a 0–4 point semi-quantitative scoring system. The criteria were defined as follows: a score of 0 represented no inflammation; a score of 1 indicated mild and focal inflammation involving less than 10% of the tissue; a score of 2 represented moderate, multifocal inflammation affecting 10–30% of the tissue area; a score of 3 denoted severe, diffuse inflammation accompanied by tissue destruction in 30–50% of the tissue; and a score of 4 indicated very severe, diffuse inflammation with extensive necrosis and abscess formation, involving more than 50% of the tissue.

### 2.6. RNA Extraction and Real-Time PCR Analysis

Total RNA was extracted by the RNA Pure Kit (Accurate Biotechnology, Changsha, China) according to the manufacturer’s instructions. The cDNA synthesis was performed using the PrimeScript RT Reagent Kit (Accurate Biotechnology, Changsha, China). Quantitative real-time PCR was executed employing SYBR ^®^ Green Pro Taq HS (Accurate Biotechnology, Changsha, China) with a StepOnePlusTM real-time PCR detection system (Applied Biosystems, Waltham, MA, USA). Given its strong stability in the expression levels and lack of differential expression observed in RNA-seq analysis results, β-actin (*ACTB*) as an internal reference gene facilitated data normalization. Subsequently, the relative gene expression values were calculated using the 2^−ΔΔCT^ method [17]. The primer sequences employed in this investigation are shown in Table 1.

### 2.7. Immunofluorescence Analysis

The mammary gland samples underwent immersion in 4% paraformaldehyde for fixation before being embedded in paraffin. The resulting blocks of mammary gland tissue were sliced into sections that measured 3 μm thick. These sections were treated with xylene to remove the paraffin, preparing them for antigen retrieval. Subsequently, the sections of the mammary gland were incubated with 5% donkey serum at room temperature for one hour, followed by overnight incubation with primary antibodies (ZO-1 and Occludin) at 4 °C. Afterward, slices of the mammary gland received staining using a goat anti-rabbit HRP-linked secondary antibody at room temperature for one hour. Finally, the sections of the mammary gland were counterstained with DAPI, and the resultant images were analyzed using a fluorescence microscope (NIKON ECLIPSE CI, Nikon, Tokyo Metropolis, Japan). Quantification of fluorescence intensity was performed in a blinded manner with respect to the experimental groups. The acquired images were analyzed using Image J (Fiji) software (version 1.8.0).

### 2.8. Transcriptome Analysis

Total RNA was isolated from 3 mammary gland samples utilizing Trizol extraction methods. The purity and concentration of extracted RNA samples were assessed using a NanoDrop 2000 spectrophotometer (Thermo Fisher Scientific, Waltham, MA, USA). Library construction using the NEBNext^®^ Ultra™ RNA Library Prep Kit for Illumina^®^ (NEB, Everett, MA, USA, Catalog #: E7530L) was performed according to manufacturer’s instructions, followed by sequencing on the Illumina novaseq 6000 (Illumina, San Diego, CA, USA).

Clean data was obtained by removing linker sequences and low-quality reads from raw data; quality assessment was conducted using FASTQC. Differential expression analysis was carried out employing DESeq2 (Bioconductor version 1.6.3), and gene expression with the threshold of *Padj* < 0.05 (followed an FDR correction) and the absolute value of |log2 fold change (FC)| ≥ 1 between two groups were classified as differentially expressed genes (DEGs).

Gene Ontology (GO) analysis encompassing biological process (BP), molecular function (MF), and cellular component (CC) was executed by GOSeq (v1.34.1) with a significant Adj. *p*. Value < 0.05. KEGG (Kyoto Encyclopedia of Genes and Genomes) was implemented by clusterProfiler 3.8.1.

### 2.9. Network Pharmacology Analysis

The active ingredients of poplar-type propolis were identified in prior study [14], including caffeic acid, *p*-coumaric acid, ferulic acid, isoferulic acid, 3,4-dimethoxycinnamic acid, cinnamic acid, pinobanksin, naringenin, quercetin, kaempferol, apigenin, pinocembrin, caffeic acid benzyl ester, 3-O-acetylpinobanksin, chrysin, caffeic acid phenethyl ester and galangin. The potential targets of the above-mentioned EECP’s active ingredients were obtained through searching TCMSP (https://tcmsp-e.com/, accessed on 16 May 2025) and SwissTargetPrediction (http://swisstargetprediction.ch/, accessed on 16 May 2025). The obtained targets were integrated, and the repeated targets were removed to obtain the final drug targets of EECP. To identify therapeutic targets related to mastitis, we searched the terms “mastitis” in GeneCards (https://www.genecards.org/, accessed on 4 September 2025) and Drugbank (https://www.drugbank.ca/, accessed on 4 September 2025). Co-targets of EECP and mastitis were identified, and used to create a protein–protein interaction (PPI) network using STRING database (http://string-db.org/, accessed on 4 September 2025) with high confidence (0.7) and drawn with Cytoscape V3.9.1 software. Additionally, gene function enrichment analysis of the co-targets was conducted using the OECloud tools (https://cloud.oebiotech.com/, accessed on 5 September 2025) to identify key signaling pathways and biological processes involved in the therapeutic effects of EECP on mastitis.

### 2.10. Molecular Docking

The 3D structure of TNF, IL-6, IL-1β, IFNG, STAT3, and CXCL8 was acquired from Uniprot (https://www.uniprot.org/, accessed on 11 September 2025), and the 3D structure of pinocembrin, pinobanksin, chrysin, CAPE and galangin were obtained from Pubchem (https://pubchem.ncbi.nlm.nih.gov/, accessed on 11 September 2025). The molecular docking was simulated using Autodock Vina. The structure of the protein–ligand complex was visualized using PyMOL Molecular Graphics System (Version 2.0 Schrodinger, Inc. New York, NY, USA).

### 2.11. Statistical Analysis

All data are expressed as the mean ± SEM. Statistical significance was indicated by asterisks and hash symbols (* *p* < 0.05, ** *p* < 0.01; # *p* < 0.05, ## *p* < 0.01). When the data test exhibited a normal distribution, the differences among the experimental groups were analyzed using a one-way ANOVA and Student–Newman–Keuls (SNK) multiple comparison test in SPSS (version 20, IBM Corp, Armonk, NY, USA). In contrast, when the data exhibited a non-normal distribution, the differences were analyzed using the Kruskal–Wallis test and Dunn’s multiple comparison test.

## 3. Results

### 3.1. Network Pharmacological Analysis of EECP in the Treatment of Mastitis

**Figure 1:**
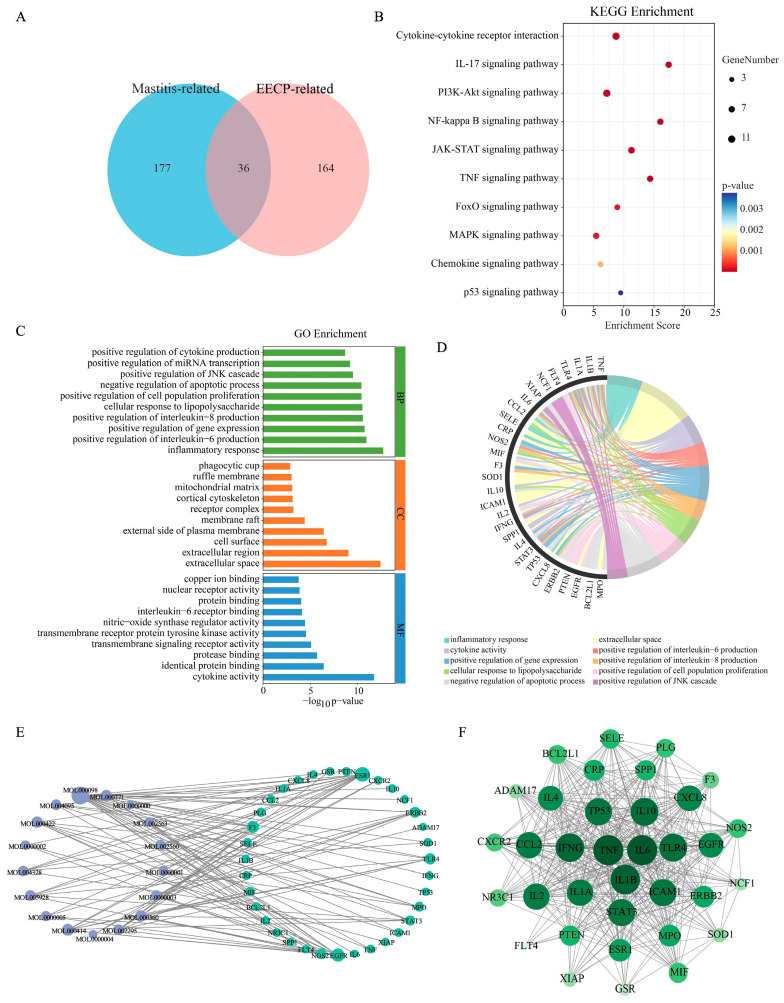
Network pharmacological analysis of EECP in the treatment of mastitis. (**A**) Venn diagram of shared targets between EECP and mastitis. (**B**) KEGG enrichment analysis. (**C**,**D**) GO enrichment analysis. (**E**) Interaction network between EECP active components and targets. (**F**) Core genes for EECP treatment of mastitis.

According to the previous study, the main components of poplar-type propolis were caffeic acid, *p*-coumaric acid, ferulic acid, isoferulic acid, 3,4-dimethoxycinnamic acid, cinnamic acid, pinobanksin, naringenin, quercetin, kaempferol, apigenin, pinocembrin, caffeic acid benzyl ester, 3-O-acetylpinobanksin, chrysin, caffeic acid phenethyl ester and galangin. To systematically investigate their mechanisms, the potential targets of these components were obtained from the TCMSP and SwissTargetPrediction databases, yielding 213 targets. Meanwhile, 200 mastitis-related genes were obtained from the GeneCards and Drugbank databases. A subsequent Venn analysis revealed 36 potential targets at the intersection of the EECP targets and mastitis-related genes (Figure 1A, Supplementary Table S1).

To elucidate the biological role of these 36 overlapping targets, the functional enrichment analysis was performed. The GO analysis indicated that in biological processes (BP), EECP is primarily associated with inflammatory response and positive regulation of interleukin-6 and -8 production. For cellular components (CC), the targets were located in the extracellular space, cell surface, and external side of plasma membrane, while their molecular functions (MF) involved cytokine activity, identical protein binding, and protease binding (Figure 1C,D). KEGG enrichment analysis showed that the top 10 signaling pathways are mainly related to physiological and pathological processes, including cell growth and proliferation (e.g., PI3K-AKT signaling pathway), immune responses (e.g., TNF, IL-17, and chemokine signaling pathway), inflammatory responses (e.g., NF-κB, JAK-STAT, and MAPK signaling pathway) (Figure 1B).

A “component–target” network was constructed using Cytoscape software, which demonstrated the close relationship between 17 components of propolis and their targets (Figure 1E). In Figure 1F, the core nodes within this network were then identified using the Degree algorithm Via the CytoHubba plug-in, which highlighted the top several targets (e.g., *TNF*, *IL6*, *IL1B*, and *IFNG*). These high-degree genes are proposed as the core targets mediating the therapeutic effects of EECP in mastitis.

### 3.2. EECP Alleviates LPS-Induced Mammary Inflammatory Response

**Figure 2:**
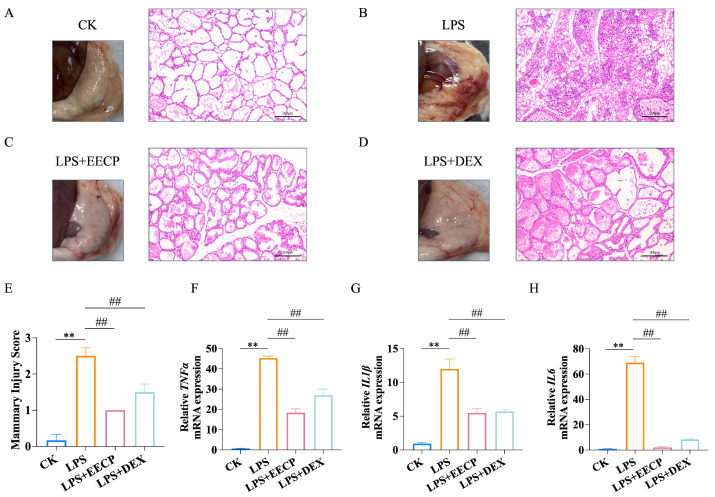
EECP Alleviates LPS-induced mammary inflammatory response in mice. (**A**–**D**) Representative H&E-stained sections of mammary glands from each group (Bar = 200 μm, *n* = 6). (**E**) Histopathological score of mammary gland. (**F**–**H**) mRNA expression levels of inflammatory cytokines in mammary tissue (*n* = 3). ** *p* < 0.01 compared with the CK group; ## *p* < 0.01 compared with the LPS group.

The results of HE staining, as illustrated in Figure 2A,B,E, revealed hyperemia, edema, and a significant level of neutrophil infiltration within the mammary tissue acini of the LPS group when compared to the CK group. However, treatment with EECP and DEX markedly alleviated these degenerative changes in the mammary gland tissues of the mice (*p* < 0.01, Figure 2C–E). In alignment with these observations, both EECP and DEX significantly suppressed the expression of proinflammatory cytokines (*TNFα*, *IL1β*, *IL6*) that were induced by LPS (*p* < 0.01, Figure 2F–H).

### 3.3. EECP Maintains Blood–Milk Barrier Integrity

**Figure 3:**
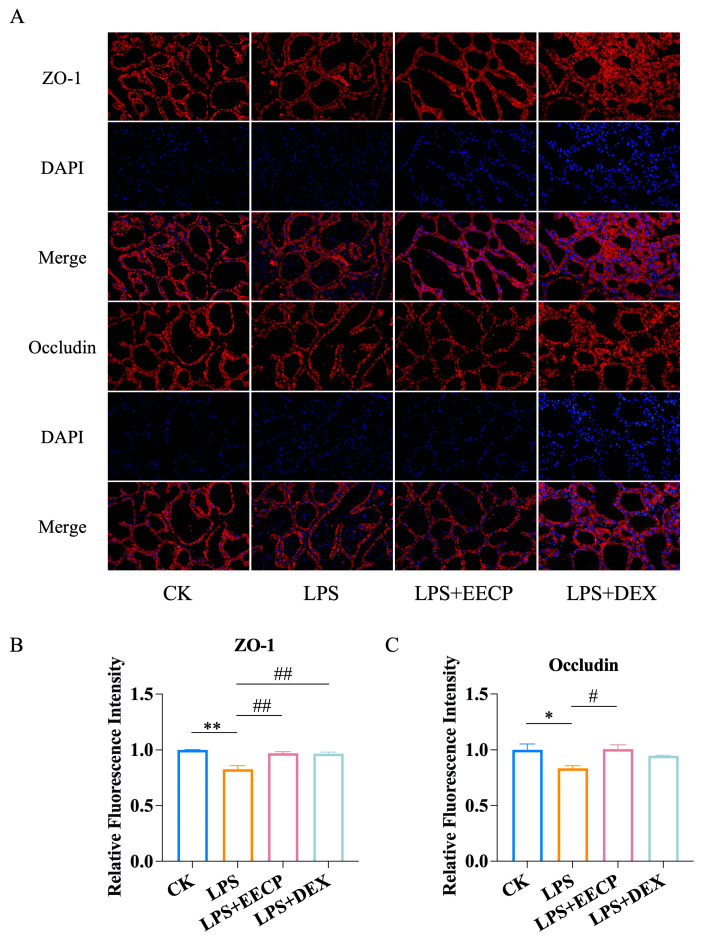
EECP maintains the integrity of the blood–milk barrier. (**A**) Representative immunofluorescence staining of ZO-1 and Occludin (Bar = 50 μm). Nuclei were counterstained with DAPI (blue). (**B**) Quantitative analysis of the mean fluorescence intensity of ZO-1 (*n* = 3). (**C**) Quantitative analysis of the mean fluorescence intensity of Occludin (*n* = 3). * *p* < 0.05 and ** *p* < 0.01 compared with the CK group; # *p* < 0.05 and ## *p* < 0.01 compared with the LPS group.

As immunofluorescence results shown in Figure 3A–C, EECP notably reversed the decreased expression levels of ZO-1 and occludin while also mitigating the disruption in tissue distribution caused by LPS in mice (*p* < 0.01). These findings suggest that EECP may preserve the integrity of tight junction complexes by enhancing the expression of tight junction proteins.

### 3.4. Transcriptomic Analysis of EECP Against Mammary Gland Injury

**Figure 4:**
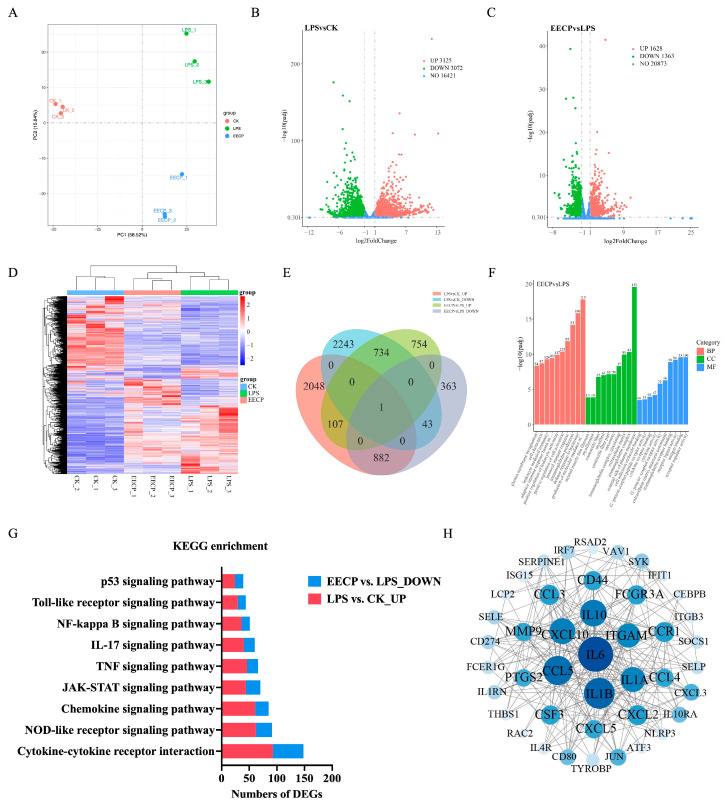
Transcriptome analysis of overlapping genes modified by EECP in the mammary gland of mice. (**A**) Principal component analysis (PCA) plot. (**B**,**C**) Volcano diagram in LPS Vs. CK and EECP Vs. LPS. (**D**) Heatmap. (**E**) Venn diagram. (**F**) GO analysis. (**G**) KEGG enrichment analysis. (**H**) Core genes for EECP treatment of mastitis.

RNA-seq analysis was conducted to compare the mammary transcriptome profiles of the CK, LPS, and LPS + EECP groups. Principal component analysis (PCA) initially confirmed a clear segregation of the transcriptomic profiles among the three groups (Figure 4A), thereby validating the robustness of our model and the distinct molecular effects of each treatment. The heatmap further corroborated the clear separation between the experimental groups and the high reproducibility of replicates within each group (Figure 4D). A volcano plot was then generated to visualize the differentially expressed genes (DEGs) (Figure 4B,C). Compared to the CK group, the LPS challenge induced significant changes in 6197 genes (3125 upregulated and 3072 downregulated). Subsequent treatment with EECP compared to the LPS group modulated 2991 genes (1628 upregulated and 1363 downregulated). GO annotation analysis (Figure 4F) showed that compared with LPS group, the DEGs in the EECP group were enriched mainly in molecular functions (MFs) involving receptor regulator activity, antigen binding, receptor ligand activity, and immunoglobulin receptor binding. The cellular component (CC) included the external side of plasma membrane, immunoglobulin complex, and extracellular matrix. Biological processes (BPs) involved production of molecular mediator of immune response, defense response to bacterium, immunoglobulin production, and humoral immune response.

KEGG enrichment analysis revealed that LPS upregulated the inflammation-related signaling pathways (e.g., TNF, JAK-STAT, IL-17 and NF-κB signaling pathways) compared to the CK group, while EECP downregulated these pathways (Figure 4G, Supplementary Table S2). This reciprocal regulation strongly suggested that the suppression of these inflammation-related signaling pathways is a key mechanism underlying the protective effect of EECP. In addition, the Venn diagram revealed that there were 882 co-expressed DEGs between the LPS Vs. CK and EECP Vs. LPS groups (Figure 4E, Supplementary Table S3), which is likely central to its therapeutic mechanism. To identify key regulatory hubs within this mechanistically relevant 882-gene set, a PPI network was constructed and analyzed using degree centrality. This identified 43 high-connectivity targets (degree > 10), with cytokines such as *IL6*, *IL1B*, and *CCL5* emerging as the core topological hubs (Figure 4H).

### 3.5. Integrated Analysis of RNA Sequencing and Network Pharmacology

**Figure 5:**
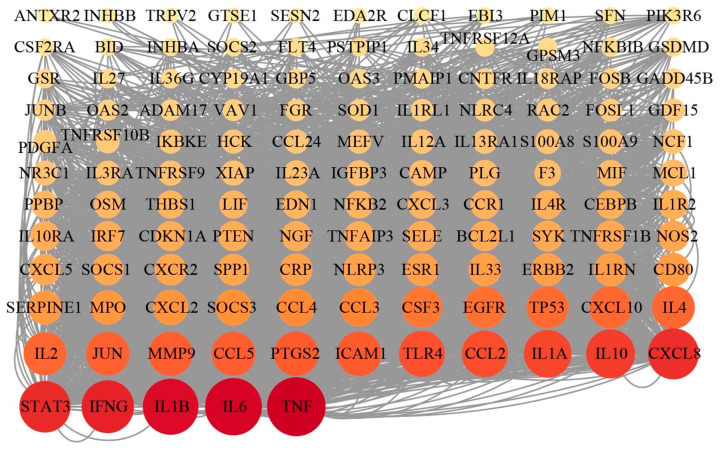
Integrated the genes from transcriptomics and network pharmacology. The nodes are colored by cluster and scaled in size according to their connectivity (degree), visually highlighting the hub targets within the network. A core set of targets—*TNF*, *IL-6*, *IL-1B*, *IFNG*, *STAT3*, and *CXCL8*—was identified.

To construct a comprehensive target landscape and minimize false negatives from either method alone, we integrated the findings from transcriptomics and network pharmacology. To focus on the most biologically relevant targets, we specifically extracted the genes belonging to the key inflammatory pathways (e.g., TNF, JAK-STAT, IL-17, and NF-κB signaling pathways) that were identified by KEGG analysis in the transcriptomic data (Figure 4G). The union of these pathway-specific genes and the targets predicted by network pharmacology yielded a final set of 127 targets for further investigation. We then ranked these 127 targets according to their degree values, a measure of network connectivity, and selected the top six as our core targets for further investigation: *TNF*, *IL6*, *IL1B*, *IFNG*, *STAT3*, and *CXCL8* (Figure 5).

### 3.6. Molecular Docking of EECP’s Key Components and Key Targets

To computationally assess the potential interaction between these six key targets and propolis, we performed molecular docking with five characteristic propolis polyphenols: pinobanksin, pinocembrin, chrysin, CAPE and galangin. All ligand–receptor combinations exhibited favorable binding energy below 0 kcal/mol (Table 2), indicating the spontaneity of the binding events. Critically, binding energy below −5 kcal/mol are generally considered indicative of stable interactions. Our results showed that all pairs met or exceeded this threshold, which provides computational evidence for their biological plausibility.

**Figure 6:**
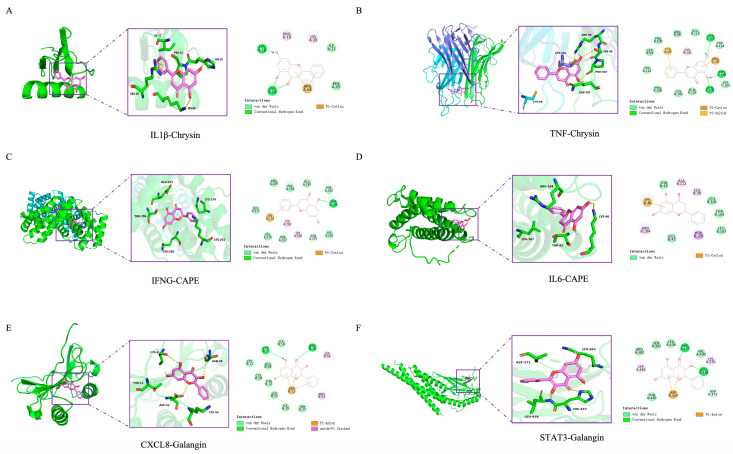
Representative 2D diagram and 3D diagram of molecular docking. (**A**) IL1β–Chrysin. (**B**) TNF–Chrysin. (**C**) IFNG-CAPE. (**D**) IL6-CAPE. (**E**) CXCL8–Galangin. (**F**) STAT3–Galangin.

Further analysis identified six optimal docking pairs with the strongest binding affinities. Chrysin showed the lowest binding energy with TNF (−7.9 kcal/mol) and IL1β (−6.7 kcal/mol) (Figure 6A,B), while CAPE exhibited the strongest binding to IL6 (−7.5 kcal/mol) and IFNG (−7.7 kcal/mol) (Figure 6C,D). Galangin bound most strongly to STAT3 (−7.0 kcal/mol) and CXCL8 (−6.7 kcal/mol) (Figure 6E,F). Beyond favorable binding energies, an analysis of the docking poses revealed specific molecular interactions that account for the high binding affinity and specificity. For instance, chrysin formed three hydrogen bonds with SER C:99, PRO C:100, and GLN C:102 of TNF-α, and its binding was further stabilized by a pi-cation stacking interaction with ARG C:98. Similarly, galangin established a hydrogen bond network with CYS B:9 and ASN B:36 of CXCL8, as well as a π-anion interaction with ASP B:52. In the case of CAPE binding to IL-6, the pi-cation stacking interaction with LYS B:46 was observed, while the complex was further embedded within a hydrophobic pocket formed by LEU B:39, LEU B:101, and PHE B:105, collectively contributing to a highly stable binding mode. This suggests that chrysin, galangin and CAPE in EECP may exert therapeutic effects on mastitis.

## 4. Discussion

Mastitis, particularly bacterial mastitis often modeled by lipopolysaccharide (LPS) challenge, is a devastating inflammatory condition of the mammary gland characterized by massive neutrophil infiltration, disruption of the blood–milk barrier, and a storm of proinflammatory cytokines [18,19]. The search for effective and natural therapeutic agents has gained significant momentum. Poplar-type propolis, rich in flavonoids (e.g., pinobanksin, pinocembrin, and chrysin) and phenolic acids (e.g., caffeic acid, *p*-coumaric acid, ferulic acid), has been renowned for centuries for its broad-spectrum biological activities, including potent anti-inflammatory and antioxidant effects [20,21]. Nevertheless, the protective effect of propolis in mastitis remains unclear, particularly with regard to the effects on specific inflammatory pathways in mammary tissue. In this study, our integrated application of network pharmacology and transcriptomics provides a systematic perspective on the anti-mastitis mechanism of poplar-type propolis. Beyond confirming its established anti-inflammatory phenotype, our study unveils a coordinated mechanism whereby EECP concurrently disrupts multiple interconnected inflammatory hubs, offering a novel paradigm for understanding its efficacy.

Seventeen main components of poplar-type propolis were selected for network pharmacology analysis based on prior quantitative profiling in our laboratory [14], which identified them as the most representative and abundant compounds. The results predicted that the key targets for these components were primarily involved in the NF-κB, JAK-STAT, PI3K-AKT, and MAPK signaling pathways. Crucially, these predicted pathways are well-established drivers of mammary inflammation [22], making them highly plausible mediators of propolis’s action. Previous studies have proven that the NF-κB and PI3K/Akt signaling pathway were promising target for therapeutic intervention to mammary inflammation. For example, phytic acid inhibits LPS-induced mammary inflammatory response by inhibiting the phosphorylation of AKT/NF-κB p65 [23]. The NF-κB pathway is a master regulator of inflammation; upon activation by LPS Via TLR4, NF-κB free p65-containing dimers translocates to the nucleus to transcribe genes for TNF-α, IL-1β, and IL-6 [24]. It is worth noting that CAPE (one of the characteristic components of propolis), is a well-established inhibitor of IκB kinase that prevents nuclear translocation of NF-κB [25]. In addition, it has been reported that apigenin alleviates LPS-induced MECs inflammatory damage by inhibiting the TLR4/NF-κB signaling pathway [26]. The PI3K/AKT pathway directly regulates LPS-induced NF-κB activation in mammary epithelial cells, and its suppression decreases the release of proinflammatory mediators such as IL-1β, IL-6, and TNF-α in LPS-stimulated mammary epithelial cells [27]. Similarly, the JAK-STAT and MAPK pathways are critical hubs for cytokine signaling and proinflammatory gene expression [28]. It has been shown that 8-methoxypsoralen treatment protects mammary epithelial cells against LPS-induced inflammatory injury by inhibition of the JAK/STAT pathways [29]. Evodiamine ameliorated LPS-induced mastitis through inhibiting the phosphorylation of ERK1/2, p38, and JNK [30]. Therefore, based on the convergence of our computational predictions with the known biology of both mastitis and EECP components, we hypothesized that the anti-mastitis effect of propolis is mediated through the coordinated suppression of this interconnected network of inflammatory pathways.

To explore the effect of EECP on mastitis, a mouse model was established Via LPS induction. Our initial findings confirmed the classical pathological features of LPS-induced mastitis, including severe hyperemia, edema, and neutrophil infiltration into the mammary tissue. EECP treatment substantially alleviated these histopathological alterations and inhibited the LPS-induced expression of the proinflammatory cytokines. Beyond mitigating inflammation, a critical finding of our study is the ability of EECP to restore the integrity of the blood–milk barrier. The blood–milk barrier is critical for maintaining mammary homeostasis [31]. The LPS-induced downregulation and disorganization of the tight junction proteins ZO-1 and occludin are hallmarks of barrier dysfunction, leading to increased permeability and compromised milk production [32]. However, EECP treatment significantly reversed this damage. This is mainly attributed to the benefit of propolis in reducing the production of proinflammatory cytokines, as it is well known that proinflammatory cytokines directly disrupt tight junction assembly [33]. Moreover, the flavonoids present in EECP have been previously reported to possess direct barrier-protective properties by modulating signaling pathways that stabilize tight junction proteins [34]. Therefore, EECP exerted a protective effect on mastitis through attenuating inflammatory responses and preserving the blood–milk barrier integrity.

Although network pharmacology analysis offered predictive insights, its virtual nature lacks biological context. To comprehensively and empirically identify the actual transcriptional changes regulated by EECP, we employed RNA-seq analysis to further verify the underlying molecular mechanism of EECP’s anti-inflammatory effects. Crucially, a key novel insight from our transcriptomic data is the synergistic downregulation of the TNF, NF-κB, JAK-STAT, and IL-17 signaling pathways. This multi-target action, predicted by network pharmacology and empirically validated by RNA-seq explains the broad-spectrum activity of this complex natural product. The PPI network of the core genes further highlighted central targets like *IL6*, *IL-1B*, and *CCL5*, a potent chemokine for leukocyte recruitment, reinforcing the central role of cytokine and chemokine signaling.

Through integrated transcriptomics and network pharmacology, we identified a core set of targets—*TNF*, *IL-6*, *IL-1B*, *IFNG*, *STAT3*, and *CXCL8*. The identification of this multi-faceted target profile is a novel finding. It positions propolis not merely as a general anti-inflammatory but as a modulator that simultaneously dampens key cytokines (TNF, IL-6, IL-1β and IFNG [35]), a critical transcription factor (STAT3), and a chemokine for immune cell recruitment (CXCL8 [36]). This holistic targeting likely underlies its ability to both resolve inflammation and preserve tissue barrier function. Pinobanksin, pinocembrin, chrysin, CAPE and galangin are characteristic compounds of poplar-type propolis, which are selected for molecular docking with six key targets. Our molecular docking results provide a structural rationale for this poly-pharmacology. This computationally supported insight moves beyond the traditional “single compound, single target” model and firmly establishes propolis as a multi-component, multi-target therapeutic system.

## 5. Conclusions

In conclusion, this study demonstrates that EECP effectively alleviates LPS-induced mastitis in mice by reducing inflammatory responses, preserving blood–milk barrier integrity, and modulating critical immune and inflammatory pathways. The integrated transcriptomics and network pharmacology analysis provides evidence that these effects are mediated through the coordinated suppression of the TNF, NF-κB, IL-17, and JAK-STAT signaling pathways, likely Via the multi-target actions of constituents such as chrysin, CAPE, and galangin. However, these promising findings warrant further validation. For example, future studies would employ primary human mammary epithelial cells to enhance translational relevance. It is crucial to establish the dose–response relationship of EECP and the pharmacokinetics of its key constituents in mammary tissue. Furthermore, mechanistic insights require protein-level validation of targets like TNF, IL-6, and STAT3, while compound-specific studies are needed to deconvolute the synergy and identify core bioactive drivers. This work provides new insights for exploring propolis as a potential natural supplement for supporting lactational health.

## Figures and Tables

**Table 1:** Primers sequences for RT-PCR.

	Gene Sense Primer (5′-3′)	Antisense Primer (5′-3′)
Mouse *TNFα*	CATCTTCTCAAAATTCGAGTGACAA	TGGGAGTAGACAAGGTACAACCC
Mouse *IL1β*	CCGTGGACCTTCCAGGATGA	GGGAACGTCACACACCAGCA
Mouse *IL6*	TAGTCCTTCCTACCCCAATTTCC	TTGGTCCTTAGCCACTCCTTC
Mouse *β-actin*	GTGACGTTGACATCCGTAAAGA	GCCGGACTCATCGTACTCC

**Table 2:** Binding energy of key EECP components and key targets.

Protein	Binding Energy (kcal/mol)				
	Pinocembrin	Pinobanksin	Chrysin	CAPE	Galangin
TNF	−7.8	−7.7	−7.9	−7.1	−7.8
IL6	−7.4	−7.2	−7.0	−7.5	−7.0
IL1β	−6.2	−6.1	−6.7	−6.2	−6.6
IFNG	−7.4	−7.6	−7.5	−7.7	−7.6
STAT3	−6.8	−6.9	−6.8	−6.2	−7.0
CXCL8	−6.5	−6.4	−6.6	−6.5	−6.7

## Data Availability

The data presented in this study are available on request from the corresponding author due to privacy.

## Supplementary Materials

The following supporting information can be downloaded at: https://www.mdpi.com/article/10.3390/nu17233683/s1, Table S1: 36 potential targets at the intersection of the EECP targets and mastitis-related genes; Table S2: Top KEGG enrichment pathways in LPS vs. CK and EECP vs. LPS groups; Table S3: 882 co-expressed DEGs between the LPS vs. CK_up and EECP vs. LPS_down groups.
